# A Comparison Between Comma Incision and Ward’s Incision in Third Molar Extraction in Terms of Postoperative Sequel

**DOI:** 10.7759/cureus.34799

**Published:** 2023-02-09

**Authors:** Aakansha Kukreja, Abhishek Balani, Vinay Kharsan, Abhishek Karan, Heena Mazhar, Arunima Awasthy, Ramanpal Singh

**Affiliations:** 1 Department of Oral and Maxillofacial Surgery, New Horizon Dental College and Research Institute, Bilaspur, IND; 2 Department of Oral Medicine and Radiology, New Horizon Dental College and Research Institute, Bilaspur, IND

**Keywords:** ward’s incision, extraction, surgery, third molar, comma incision

## Abstract

Aim

We aim to look at the differences between the standard Ward’s incision and the comma-shaped incision and how they affect complications after surgery to remove an impacted mandibular third molar.

Materials and methods

Mandibular third molars had to be carefully extracted from a total of 40 patients who were randomly divided into two groups of 20 patients each. At first, patients were evaluated before surgery. In group A, a standard Ward’s incision was made, and in group B, a comma incision was made to match the mucoperiosteal fold. Afterward, the impacted third molars were carefully removed. The evaluation criteria for pain, swelling, lockjaw, and healing of wounds were done before surgery, after three hours, and on the first, third, and seventh day after surgery.

Result

The pain scores that were recorded right after surgery, three hours later, and on days 1, 3, and 7 in the surgical area with comma-shaped incision were all lower than the pain scores that were recorded in the area where standard incisions were made. Enlarging was less with comma entry point than with standard Ward’s incision. After surgery, there was a big difference between the two entry points in how the mouth opened and how the wounds were fixed. These findings showed that the comma incision is better than the standard Ward’s incision when it comes to pain, enlargement, lockjaw, and healing of wounds.

Conclusion

The study results showed that the comma-shaped incision was better than the traditional method (Ward’s incision) because there were fewer problems after surgery.

## Introduction

Up to 90% of people have third molars, and 33% have at least one that is impacted [1]. The most frequent type of minor oral surgery is the surgical removal of impacted third molars. The third molar in the mandible is most susceptible to being impacted. Most studies have shown that there is no sexual preference. Some studies, though, have shown that it happens more often in women than in men [1]. Surgical removal involves manipulating both hard and soft tissues. It is often followed by a wide range of problems [2]. The flap must be able to move far enough away from the planned osteotomies and odontectomies so that the affected area can be seen well, and surgery can be done. When making the flap, important parts of the body such as the end of the second molar, the lingual nerve, and the buccinator muscle should be taken into account. The flap should also have a wide base to make sure blood flow is good [2]. Dependable standards determine the entry point and fold configuration for any surgery. As much as possible, entry point lines should not cross important muscle or ligament additions or lie over possible bone deformations.

A conventional Ward’s cut is made distal to the subsequent molar, and it extends over the alveolar apex (if the tooth is completely embedded) or along the buccal gingival sulcus of the third molar up to the distal conveying cut, which is made by slicing the external sloping edge of the third molar into the buccal mucosa. For better openness, the front delivering entry point should be moved up to the end of the first molar if necessary.

An incision resembling a comma is performed forwardly from the most acute mark of this extended vestibular reflection back to the farthest limit of the prior second molar. An incision is created beneath the next tooth and brought to a point. From there, an impeccable twist will bring it up to the gingival apex of the next molar at the distobuccal line. A hole-like incision is made around the tip of the third molar to create the entrance [3].

The end of a typical Ward’s incision, which is usually used to carefully remove infected mandibular third molars, comes close to or even cuts through the temporalis ligament, which is a common cause of lockjaw after surgery. The fold usually hides the bone loss that happens after the impacted tooth is taken out, which can lead to a slower recovery, pain, and infection.

The comma-shaped cut lets a distolingual-based flap be reflected, which shows the whole third molar region. The next clean area makes it very easy for a dentist to use the traditional buccal bone removal system or the lingual split system. After the affected tooth is taken out, the fold can be repositioned and fixed with a couple of stitches. The injury does not come close to the retromolar cushion or the addition of the temporalis muscle ligament, nor does it come close to the resulting bone deformity [3,4].

## Materials and methods

Methodology

Forty patients, ranging in age from 18 to 60, were referred to the Department of Oral and Maxillofacial Surgery at New Horizon Dental College and Research Institute, Sakri, Bilaspur, Chhattisgarh, India, with institutional review board (IRB) number NHDCRI/2022/11, for careful extraction of infected mandibular third molars.

Surgical method

Forty individuals with impacted third molars in the mandible were recruited for the research and divided evenly between two groups of 20. In group A, the mandibular third molars were extracted using a standard Ward’s incision. In group B, the mandibular third molars were extracted using a comma incision.

In all sterile conditions, the patients were anesthetized using 2 mL of 2% lignocaine with adrenaline. We performed an inferior alveolar nerve, lingual nerve, and long buccal nerve block (1:200,000).

Group A: Ward’s Incision Group

As shown by Ward, a sulcular entry site was created by extending the mesiobuccal border of the second or first molar to the distal surface to obtain access to the tooth that was impacted. They allow the interdental papilla between the second and third teeth to be the mesial section location because it is more aesthetically pleasing. To facilitate delivery, a horizontal incision was made along the mandibular ramus, either at the midpoint or at the distal portion of the distolingual cusp of the following molar, depending on tissue inclusion and impaction depth.

Group B: Comma Incision Group

**Figure 1:**
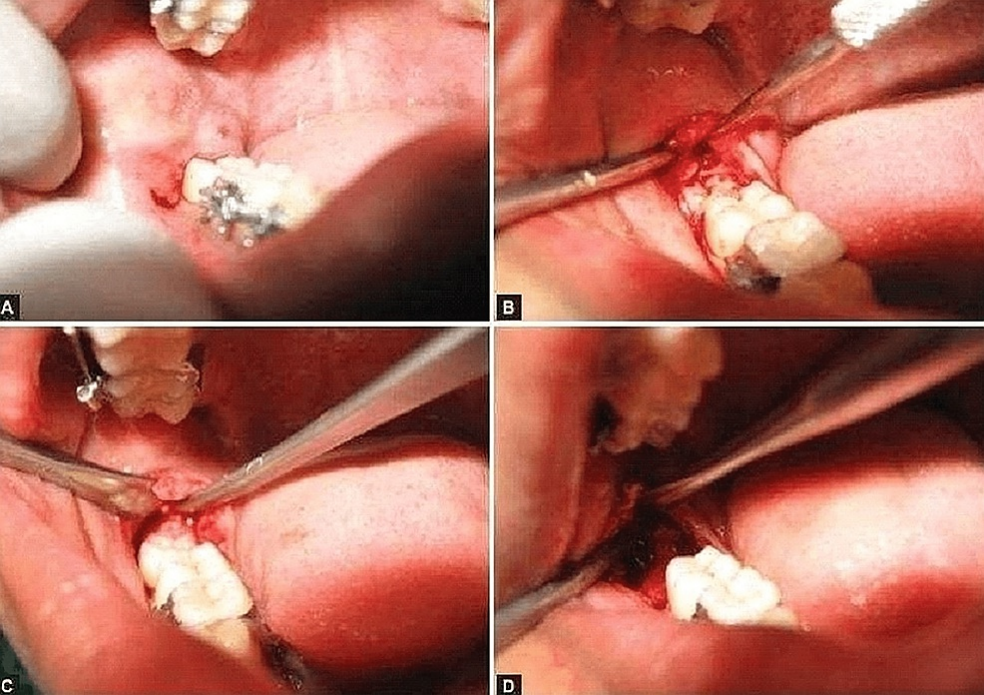
Comma incision for the procedure of disimpaction

A buccal incision called the “comma” was used in this group (Figure 1). It began at the base of the elongated vestibular reflection and extended back to the area below the previous second molar. The incision was created in a forward direction all the way to a position beneath the next molar, from whence it will be wonderfully bowed up to meet the gingival top at the distobuccal line point of the succeeding molar. Next, a crevicular entrance point will be executed around the distal viewpoint of the succeeding molar. After the incision was established, the thick mucoperiosteal fold encompassing the third molar area was elevated and retracted. Buccal guttering was done using the straight diamond bur numbers 702 and 703, and the round diamond bur number 8. The tooth will be extracted using lifts and forceps in a saline solution, and then, the fold will be realigned and closed using 3-0 black braided silk sutures. All patients will be given a five-day course of antibiotics consisting of amoxicillin 500 mg, metronidazole 400 mg, and diclofenac potassium 50 mg tablets. The patients will be kept on follow-up on the first, third, and seventh postoperative days, and all parameters (pain, swelling, trismus, and wound healing) will be assessed.

Patients with a third tooth in the mandible that has been impacted who belong to the ASA 1 group (A and B, class 1 and 2, and patients willing to report for follow-up study) and were between 18 and 60 years were included in the present study. Patients with serious medical conditions who were in the ASA 2-ASA 4 group (pregnant, class 3, substance abuse and alcoholism, refusal to provide informed permission, presence of acute infection at the injection site, and long-term use of central nervous system (CNS) depressants or antidepressants) were excluded.

Statistical analysis

The visual analog scale (VAS) was used to measure the degree of pain, from 0 to 10. Superoinferior growth was assessed from the ala tragus line to the lowered mandibular border, and anteroposterior growth was measured from the corner of the mouth to the tragus of the ear [5]. The indicator of trismus (according to Wood and Branco [6]) is the greatest gap between the two incisors. Recovery after an injury is calculated using the clinical models for effective healing by Holland and Hindle [7]. Socket healing was determined to be primary if a blunt probe could not be inserted into the socket via a mucosal defect or secondary if a mucosal defect did exist.

Assessment is done using the Statistical Package for the Social Sciences (SPSS) version 28.0.1.1 (IBM SPSS Statistics, Armonk, NY, USA) by applying Student’s t-test and Mann-Whitney U test. Changes in discomfort, edema, and trismus ratings were examined over time using a repeated measures method. As a rule of thumb, a P value of less than 0.05 was considered significant in the aforementioned tests.

## Results

Of the participants, 20% were in the 18-25 age range. Group A consisted of 5% of the study population, whereas group B included 15% of the research population. Participants between the ages of 26 and 30 made up 32.5% of the study’s total population. About 15% of the study population was assigned to group A, whereas 17.5% was assigned to group B. Furthermore, 31- to 35-year-olds made up approximately 35% of the study population. Group A included 22.5% of the participants, whereas group B included 12.5% of the same age range. About one in every 15 participants was between the ages of 36 and 40. In this age range, 5% of the participants were in group B, whereas 7.5% were in group A (Table 1).

**Table 1:** Distribution of study subjects according to age group

Age group	Number		Percentage	
	Group A	Group B	Group A	Group B
18-25	2	6	5	15
26-30	6	7	15	17.5
31-35	9	5	22.5	12.5
36-40	3	2	7.5	5

Participants in both groups were evenly divided between males and females, with males making up 30% of group A and females making up 20% of the whole of group B. The 10-point visual analog scale (VAS) was used to measure suffering. Before surgery, the VAS scores were 0.1±0.002 in group A and 0.2±0.001 in group B on average. The VAS scores (mean±standard deviation (SD)) for group A ranged from 5.43±1.21 to 4.64±1.31, to 2.91±0.03, to 0.91 ±0.01 after three hours, 24 hours, 72 hours, and one week, respectively. The VAS scores (mean±SD) right after surgery, 24 hours after, 72 hours after, and after one week in group B were 5.2±1.11, 3.36±1.21, 1.47±0.04, and 0.16± 0.01, respectively. Significant intragroup differences were seen, with pain decreasing with increasing time after surgery. It was revealed that the outcome postoperatively was decreased in group B participants (Table 2).

**Table 2:** Pain details in both groups

Duration	Group A	Group B	P value
Preoperative	0.1±0.002	0.2±0.001	0.76
Immediate postoperative (three hours)	5.43±1.21	5.21±1.11	0.03
First postoperative day (24 hours)	4.64±1.31	3.36±1.21	0.02
Third postoperative day (72 hours)	2.91±0.03	1.47±0.04	0.02
Seventh postoperative day (one week)	0.91±0.01	0.16± 0.01	0.01
P value	0.001	0.001	

Trismus was assessed by estimating the maximum interincisal distance using a divider and a ruler [6]. The preoperative interincisal mouth opening distance (mean±SD) was 37.32±1.12 mm in group A and 38.45±0.98 mm in group B. The postoperative interincisal mouth opening distances (mean±SD) at three hours, 24 hours, 72 hours, and one week were 33.21±1.33 mm, 16.34±1.23 mm, 11.32±1.10 mm, and 28.45±1.20 mm, respectively, in group A. The postoperative interincisal mouth opening distances (mean±SD) at immediate postoperative three hours, 24 hours, 72 hours, and one week were 36.56±1.12 mm, 26.24±1.11 mm, 18.23±1.11 mm, and 38.34±1.11, respectively, in group B. The intragroup contrasts were huge genuinely. There was expansion at 24 hours follow-up when contrasted with immediate postoperative three hours follow-up in the two groups. The trismus further expanded at 72 hours follow-up in contrast with 24 hours follow-up in the two groups. The trismus diminished at seven days follow-up in the two groups. The intergroup factual contrasts were likewise huge measurably. It was found that the enlarging result postoperatively was less in group B (Table 3).

**Table 3:** Postoperative trismus (interincisal mouth opening) outcomes in both groups

Duration	Group A	Group B	P value
Preoperative	37.32±1.12	38.45±0.98	0.89
Immediate postoperative (three hours)	33.21±1.33	36.56±1.12	0.01
First postoperative day (24 hours)	16.34±1.23	26.24±1.11	0.04
Third postoperative day (72 hours)	11.32±1.10	18.23±1.11	0.02
Seventh postoperative day (one week)	28.45±1.20	38.34±1.11	0.01
P value	0.001	0.001	

The clinical criteria for successful wound healing according to Holland and Hindle [7] were used to assess wound closure. Among those in group A, 92.23% were deemed to have achieved sufficient healing, whereas among those in group B, 96.46% did so. Wound healing was much better in group B, indicating a statistically significant difference between the groups. Furthermore, there were statistically significant variations among groups, with recovery progressing more rapidly as time passed after surgery (Table 4).

**Table 4:** Postoperative wound healing in both groups ^a^Intergroup ^b^Intragroup

Duration	Group A	Group B	P value^a^
Seventh postoperative day	92.23	96.46	0.001
P value^b^	0.001	0.001	

## Discussion

The severity of postoperative problems may be affected by a number of factors, one of which is the form of the incision [8]. This has led us to examine two alternative incision designs, one based on the traditional process for a third molar disimpaction procedure, known as Ward’s incision, and the other based on a distolingual-based comma-shaped incision. Extraction of the impacted third molars is usually accompanied by discomfort, swelling, and trismus. The pain was measured using a visual analog scale (VAS) that ranged from 1 to 10.

Similar to the findings of Neelkandan et al. [9] and Nageshwar [1], these new findings are also worth noting. A comparative research was conducted that identified findings similar to what we found in our evaluation. However, Erdogan et al. [10] obtained results not similar to those of the present study because they found no appreciable difference in discomfort between the two procedures. In addition, the frequency and intensity of symptoms might vary widely from person to person [5]. There are a number of variables that might affect one’s perception of pain, and several ways of measuring pain have been described in published research [11]. Assuming the repair process proceeds normally, the level of disruption should gradually lessen over the next several days. Pain ratings were significantly lower in the comma incision group compared to Ward’s incision group on day 1 after surgery and were also lower on days 3 and 7, but these differences were not statistically significant. This might be because less tissue is damaged than at traditional entrance locations.

We measured the affected area in two dimensions for this analysis: the anterior-posterior (from the corner of the mouth to the tragus of the ear) and the superior-inferior (from the ala tragus line to the lower boundary of the jaw). The divide between the two groups was palpable. At the 24-hour follow-up, in both groups, edema appeared more rapidly than in the preceding three hours. The edema increased significantly over the 24- and 72-hour follow-up periods. Edema had diminished in both groups at the seven-day follow-up.

The differences between the groups were also significantly measurable. Participants in group B had significantly less postoperative edema compared to those in group A. After surgery, the improved incision design reduces the chance of problems including edema, according to the research. Two different flap designs for mandibular third molar surgery were compared for their effects on postoperative complications [10]. No similarities were found between the results of this research and those of the current investigation. The findings of the current research are consistent with those of Neelkandan et al. [9] and Nageshwar et al. [1]. For this analysis, we define “enlargement” as the horizontal distance between the tragus and the jaw’s soft tissue pogonion [12]. When compared to other methods of measuring swelling, this one is quick, painless, and inexpensive. Several studies have looked at the timing of edema after extraction of the third mandibular tooth. After surgery, edema begins to increase quickly and reaches its peak between 24 and 72 hours later [13].

At seven days of follow-up, edema had reduced in both groups. As expected, there were statistically significant differences between the groups as well. Group B saw reduced postoperative edema compared to group A. Overall, the edema reported by the comma incision group was smaller than that reported by Ward’s incision group. Together, our findings corroborate those of Neelkandan et al. [9] and Nageshwar et al. [1]. Using a divider and a ruler, as advocated by Wood and Branco, the greatest interincisal distance was calculated to evaluate lockjaw in the current study. True significance was added by contrasting within-group factors. In all groups, lockjaw worsened during the 24-hour follow-up stage compared to the three-hour follow-up stage. When comparing the two groups’ progress at 24 and 72 hours after the initial injury, lockjaw showed more growth at the 72-hour mark.

Suarez-Cunqueiro et al. [14], Neelkandan et al. [9], Nageshwar et al. [1], and Jakse et al. [15] conducted studies along similar lines to ours and found comparable outcomes. The results of the research by Erdogan et al. [10] did not identify a statistically significant difference between the two procedures for postoperative trismus; hence, it is not comparable to our research.

The clinical criteria for successful wound healing provided by Holland and [7] were used to assess wound closure. Among those in group A, 92.23% were deemed to have achieved sufficient healing, whereas among those in group B, 96.46% did so. Wound healing was much better in group B, indicating a statistically significant difference between the groups. Furthermore, there were statistically significant variations among groups, with recovery progressing more rapidly as time passed after surgery. Both Jakse et al. [15] and Suarez-Cunqueiro et al. [14] support the idea that incision impacts essential injury recuperating in a third molar disimpaction procedure.

The impact of two different flap designs on pain and swelling after the surgical extraction of impacted mandibular third molars was studied by Yazdani et al. [16] in 2014. The flap design had no significant effect on pain and swelling after the surgical extraction of impacted mandibular third molars. Desai et al. [3] compared two incision designs for the surgical removal of impacted mandibular third molars in a prospective comparative analysis. No significant differences were noticed between the groups in terms of visibility, accessibility, excessive bleeding during surgery, healing of the flap, sensitivity of adjacent teeth, and dry socket. A statistically significant difference was noticed in postoperative hematoma, wound gaping, and distal pocket in the neighboring tooth, which was significant in Ward’s triangle incision group in comparison to Koener’s envelope incision group.

The surgical removal of third molars is one of the most frequently performed procedures in the oral and maxillofacial practice to prevent or treat a variety of pathoses originating from impacted teeth [1,2]. The removal of such teeth requires a sound understanding of surgical principles along with patient management skills. It must be performed properly to allow expeditious and atraumatic removal of teeth embedded in a relatively atraumatic area of the oral cavity. Although it is a minor surgical procedure, its relation to the adjacent teeth, soft tissues, and neurovascular bundle makes it a complex procedure. Surgical removal involves the manipulation of both soft and hard tissues, so it is usually associated with a number of postoperative complications [17,18]. Therefore, reducing the incidence of these complications becomes imperative, which is possible only with a thorough knowledge of the various factors affecting them.

One of the factors influencing the postoperative outcome following third molar surgery is the incision and flap design. The flap design is important not only to allow optimal visibility and access to the impacted tooth but also for subsequent healing of the surgically created defect [19]. The distal leg of the incisions conventionally made to access impacted mandibular molars comes close to or even cuts across the insertion of the temporalis tendon. It also commonly lies over bone defects formed after the removal of the tooth. This could be responsible, at least in part, for the occurrence of these complications. This, therefore, is enough reason to consider alternative incision and flap designs [20].

The limitations of the study were the limited sample size, and only a few parameters and two incisions were tested.

## Conclusions

The conclusion of the study is that a comma incision is better than the traditional Ward’s incision in terms of postoperative complications as few difficulties are encountered with comma incision, which include inadequate accessibility and more time consumption. The study could have been more accurate if we had added more parameters such as the periodontal pocket depth measurement of the adjacent second molar and the incidence of dry socket postoperatively.
